# Refractory Periorbital Necrobiotic Xanthogranuloma Treated With Plasma Cell‐Directed Therapy

**DOI:** 10.1002/jha2.70223

**Published:** 2026-01-19

**Authors:** Aaron Trando, Ah‐Reum Jeong, Aaron M. Goodman

**Affiliations:** ^1^ Department of Medicine University of California San Diego La Jolla California USA; ^2^ Division of Blood and Marrow Transplantation University of California San Diego La Jolla California USA

1

A 57‐year‐old female with a reported history of isolated neutropenia presented with 3 years of progressive bilateral vision loss and 4 months of a rapidly growing plaque on her left eyelid (Figure 1A). MRI of the orbits revealed a 1.4 cm left superomedial preseptal mass with retroseptal extension (Figure 1B). On pathological analysis after left anterior orbitotomy, biopsy samples demonstrated subepithelial histiocytic inflammation with band‐like zones of necrobiosis comprised of xanthic epithelioid histiocytes, including multinucleated giant cells. Such histological features resembled those conveyed in Figures 3 and 5 of the report published by Wood et al. [1] and were consistent with necrobiotic xanthogranuloma (NXG). NXG is a rare and chronic type of non‐Langerhans cell histiocytosis commonly associated with paraproteinemia in up to 80% of patients, representing monoclonal gammopathy of clinical significance [2]. Figure 1

**FIGURE 1:**
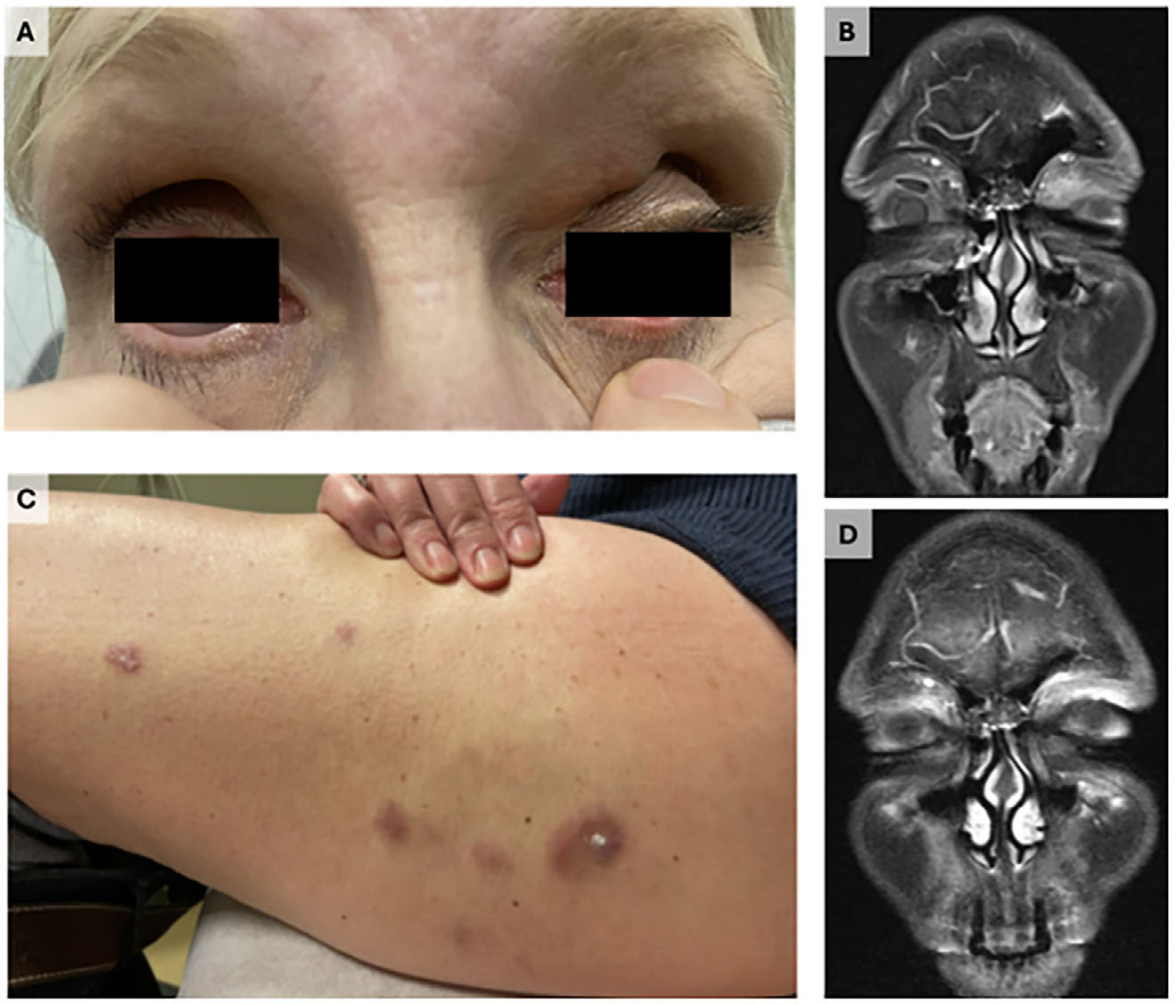
Physical examination demonstrated necrobiotic xanthogranuloma skin lesions of the patient's left eyelid (A) and left upper thigh (C). MRI of the orbits demonstrated a left superomedial preseptal mass (B) that resolved after treatment with plasma cell‐directed therapy (D).

Laboratory evaluation was notable for an IgG lambda M‐spike. Bone marrow biopsy revealed a lambda‐restricted plasma cell population. Imaging and biopsies of her liver and left lower extremity skin lesions confirmed further involvement of NXG (Figure 1C). The patient went on to receive numerous treatment regimens, including oral steroids (a 5‐week taper of prednisone 60 mg), IVIG (4 monthly treatments), rituximab (4 weekly treatments), and orbital radiation (24 gray/12 fractions). None of these therapies induced a clinical response, and so plasma cell‐directed therapy was attempted.

She subsequently received monthly cycles of daratumumab (1800 mg monthly), lenalidomide (25 mg Days 1–21), and dexamethasone (20 mg weekly that was then tapered) (DRd). After 10 months of DRd/DR therapy, the patient's M‐spike has decreased, her cutaneous lesions have improved, and no focal orbital masses were identified on repeat MRI (Figure 1D). To our knowledge, this is the first reported response of NXG to a daratumumab‐containing regimen. As the granulomatous pathophysiology of NXG may be driven by the presence of a concurrent monoclonal paraprotein, plasma cell‐directed therapies should be considered in cases refractory to conventional treatment options like alkylating agents, steroids, or IVIG [3].

## Author Contributions

A.T., A.‐R.J., and A.G. all cared for the patient and assisted with data collection. A.T. wrote the manuscript and prepared the images. A.‐R.J. and A.G. reviewed the manuscript.

## Funding

The authors have nothing to report.

## Conflicts of Interest

The authors declare no conflicts of interest.

## Ethics Statement

This case report was conducted in accordance with the Declaration of Helsinki. The collection and evaluation of all protected health information was performed in a Health Insurance Portability and Accountability Act (HIPAA)‐compliant manner.

## Consent

Written informed consent was obtained from the patient, including permission for publication of images.

## Data Availability

The data that support the findings of this study are available from the corresponding author upon reasonable request.
